# [Retracted] Inhibition of tankyrase 1 in human gastric cancer cells enhances telomere shortening by telomerase inhibitors

**DOI:** 10.3892/or.2026.9101

**Published:** 2026-03-16

**Authors:** Hao Zhang, Meng-Hua Yang, Jing-Jing Zhao, Ling Chen, Song-Tao Yu, Xu-Dong Tang, Dian-Chun Fang, Shi-Ming Yang

Oncol Rep 24: 1059–1065, 2010; DOI: 10.3892/or_00000955

Following the publication of the above paper, it was drawn to the Editor's attention by a concerned reader that, for the bottom panel of blots in Fig. 2c on p. 1062 showing the expression of TP1 mRNA, the data in lanes 1–4 bore a remarkable similarity to those featured in lanes 5–8, even though the lanes were reported to show the results of differently performed experiments. Moreover, upon inspecting the data further in the Editorial Office, there were possible anomalies associated with the western blots in Fig. 2A, including uneven alignments of protein bands and potential breaks in the continuity of certain of the gels.

The authors were contacted by the Editorial Office to ask them to provide an explanation for these apparent anomalies in the presentation of the data in this paper; however, up to this time, no response from them has been forthcoming. Therefore, the Editor of *Oncology Reports* has decided that this paper should be retracted from the Journal on account of a lack of confidence in the presented data. The Editor apologizes to the readership for any inconvenience caused.

